# Extracellular overexpression of recombinant *Thermobifida fusca *cutinase by alpha-hemolysin secretion system in *E. coli *BL21(DE3)

**DOI:** 10.1186/1475-2859-11-8

**Published:** 2012-01-12

**Authors:** Lingqia Su, Sheng Chen, Li Yi, Ronald W Woodard, Jian Chen, Jing Wu

**Affiliations:** 1 State Key Laboratory of Food Science and Technology, Jiangnan University, 1800 Lihu Ave, Wuxi, Jiangsu 214122, China; 2 School of Biotechnology and Key Laboratory of Industrial Biotechnology, Ministry of Education, Jiangnan University, 1800 Lihu Ave, Wuxi, Jiangsu 214122, China; 3 Department of Medicinal Chemistry, University of Michigan, Ann Arbor, Michigan 48109, USA

**Keywords:** alpha-hemolysin secretion pathway, cutinase, protein secretion, extracellular production

## Abstract

**Background:**

Extracellular expression of proteins has an absolute advantage in a large-scale industrial production. In our previous study, *Thermobifida fusca *cutinase, an enzyme mainly utilized in textile industry, was expressed via type II secretory system in *Escherichia coli *BL21(DE3), and it was found that parts of the expressed protein was accumulated in the periplasmic space. Due to the fact that alpha-hemolysin secretion system can export target proteins directly from cytoplasm across both cell membrane of *E. coli *to the culture medium, thus in the present study we investigated the expression of cutinase using this alpha-hemolysin secretion system.

**Results:**

*T. fusca *cutinase was fused with the specific signal peptide of alpha-hemolysin scretion system and expressed in *E. coli *BL21(DE3). In addition, HlyB and HlyD, strain-specific translocation components of alpha-hemolysin secretion system, were coexpressed to facilitate the enzyme expression. The cultivation of this engineered cell showed that cutinase activity in the culture medium reached 334 U/ml, which is 2.5 times that from type II secretion pathway under the same culture condition. The recombinant cutinase was further purified. Biochemical characterization of purified enzyme, which had an α-hemolysin secretion pathway signal peptide attached, had substrate specificity, pH and temperature profile, as well as application capability in bioscouring similar to that of wild-type cutinase.

**Conclusions:**

In the present study, *T. fusca *cutinase was successfully secreted to the culture media by α-hemolysin secretion system. This is the first report of cutinase being efficiently secreted by this pathway. Due to the limited cases of successful expression of industrial enzyme by *E. coli *α-hemolysin secretion system, our study further explored the utilization of this pathway in industrial enzymes.

## Background

Generally, extracellular expression of proteins has an absolute advantage in a large-scale industrial production. *Escherichia coli*, the most widely used host, has five protein secretion pathways, in which, the vast majority of recombinant proteins are secreted using the SecB-dependent type II pathway. In this process, the pre-protein is first transferred across the inner membrane, folded in the periplasm, and then secreted into the culture medium, usually by nonspecific periplasmic leakage [1]. Due to this two-step process, the recombinant protein is sometimes partially located in the extracellular medium and partially in the periplasm, affecting the total extracellular yields [2,3].

By contrast, type I secretion systems (TISS) export their native passenger proteins or recombinant proteins/peptides such as repeats-in-toxin (RTX) proteins, proteases and lipases directly into the culture medium without accumulation in the periplasmic space [4-9]. The best-characterized and most widely used TISS is the α-hemolysin (HlyA) secretion pathway from uropathogenic *E. coli *[10]. The secretory machinery of this pathway consists of three components: HlyB, an ATP-binding cassette (ABC transporter); HlyD, a membrane fusion protein; and TolC, an outer membrane protein. The three components can form a trans-membrane channel connecting both inner and outer membranes of cell, through which HlyA can be transferred directly to the extracellular medium. In this pathway, the signal peptide, which is located in the C-terminus of HlyA, is not cleaved after translocation and remains in the target protein as the final product [11].

In addition to HlyA, proteins ranging in size from 50 to over 4000 amino acids, with C-terminally fused HlyA signal sequence (HlyAs), can also be recognized and secreted via the HlyB-HlyD-TolC translocator [1,11]. The α-hemolysin secretion pathway has mainly been reported in uropathogenic *E. coli*. HlyB and HlyD have been generally considered strain specific proteins, TolC, in contrast, is a component of multiple trans-membrane systems in many microorganisms. Thus, co-expression of HlyB/D is often performed to facilitate the extracellular translocation of proteins utilizing the α-hemolysin secretion pathway during expression in host strains other than uropathogenic *E. coli *[12]. So far, this approach has most often been applied to the expression of proteins involved in immunological and vaccine research, especially in the recombinant antigen presentation through live-attenuated bacterial vaccine strains [11,13-15], there were only several cases of successful extracellular expression of industrial enzymes, such as lipase [16], protease [15], cyclodextrin glucanotransferase [17], alkaline phosphatase [18]. Recent reports about α-hemolysin secretion pathway have focused on the effects of translated protein folding rate and mutagenesis of HlyAs, HlyB or HlyD on the efficiency of translocation [15,17,19].

Cutinase is a multi-functional esterase, which shows hydrolytic activity (cutin and a variety of soluble synthetic esters, insoluble triglycerides and polyesters), synthetic activity and transester activity [20-28]. Therefore, it is an important industrial enzyme. Especially, due to the capacity of hydrolyzing the cutin structure in the cuticle of cotton fiber, it has potential application in environmentally friendly bioscouring [29]. Previously, cutinase from *Thermobifida fusca *was cloned and extracellularly expressed in *E. coli *BL21(DE3) through type II secretory system in our laboratory [30]. Large amounts of cutinase were found in the periplasmic space [22]. In the present study, we showed that the *T. fusca *cutinase could be efficiently secreted to the external medium through α-hemolysin secretion pathway without a periplasmic intermediate. In addition, detailed characterization showed that the recombinant enzyme, even with c-terminal signal peptide attached, has similar properties to that of wild-type cutinase.

## Results and discussion

### Construction of recombinant plasmids

In order to use *E. coli *BL21(DE3) to express recombinant protein into the growth medium through the α-hemolysin secretion pathway, the α-hemolysin specific signal peptide HlyAs, which corresponds to residues 965-1024 of α-hemolysin, was fused to the C-terminus of *T. fusca *cutinase by overlapping PCR. The PCR products were cloned into the T7-driven expression vector pET-20b(+), resulting in the recombinant plasmids cutinase-HlyAs/pET-20b(+). In addition, the genes encoding the components of translocator, HlyB and HlyD, were amplified together by PCR using *E. coli *CFT073 genomic DNA as template, and cloned into the expression vector pSTV28, resulting in HlyBD/pSTV28.

### Extracellular secretion of recombinant cutinase-HlyAs

**Figure 1:**
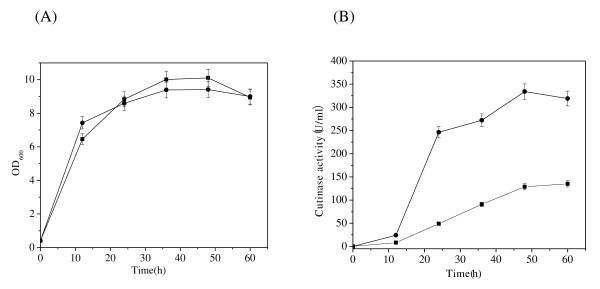
**Cell growth(A) and the production of recombinant cutinase(B)**. (●) type I secretion pathway; (■) type II secretion pathway. The error bars show the standard deviations from three measurements.

To create an extracellular expression system for cutinase via the α-hemolysin secretion pathway, the *E. coli *BL21(DE3) strain was co-transformed with two plasmids: cutinase-HlyAs/pET-20b(+) and HlyBD/pSTV28. Since the two plasmids contain different replication origins, they have capability of co-existing in one bacterial cell. When subsequently grown in TB medium, the cutinase activity in the culture medium reached 334 U/ml at 48 h (Figure 1B), which is 2.5 fold to that of the culture by a recombinant strain constructed previously in which cutinase was secreted through *E. coli *secB-dependent type II pathway [30]. The protein concentration of expressed enzyme was 1.5 mg/mL, which represents the highest yield of cutinase ever reported in the culture medium [2,31,32]. No significant difference in cell growth was observed between these two engineered strains (Figure 1A).

### Purification of cutinase-HlyAs

**Figure 2:**
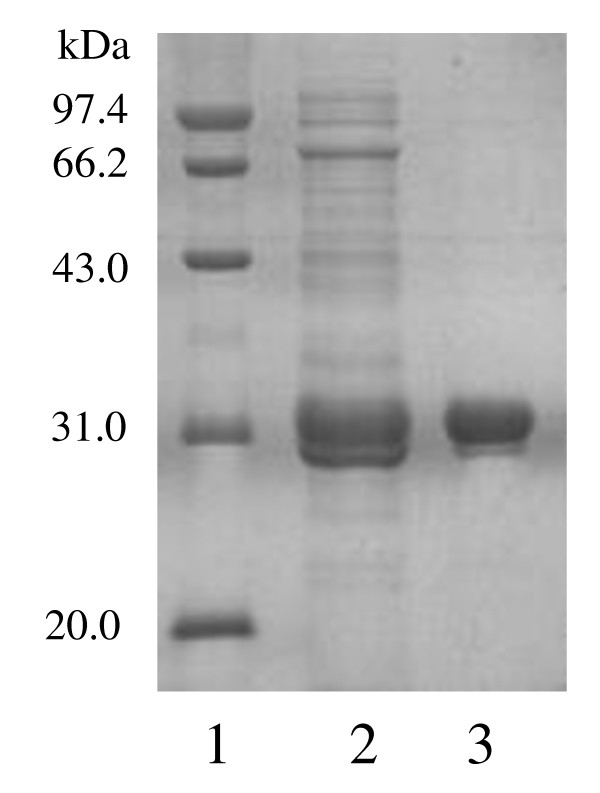
**SDS-PAGE analysis of the purification of cutinase-HlyAs in *E. coli***. Lane 1, molecular mass standard protein; Lane 2, supernatant of cutinase-HlyAs; and Lane 3, purified cutinase-HlyAs.

In α-hemolysin secretion pathway, the signal peptide (HlyAs) will not be cleaved after translocation [12] and remained in the final protein product, which is cutinase-HlyAs in our case. Cutinase-HlyAs was purified from culture supernatant by ammonium sulfate precipitation and anion exchange (DEAE-Sepharose, monoQ) chromatography. The purification process is summarized in Table 1. The purified cutinase-HlyAs was determined to be homogeneous by SDS-PAGE (Figure 2) and exhibited a specific activity of 446.2 U/mg using the substrate pNPB, which is similar to that of cutinase activity [30].

**Table 1:** Summary of the purification of cutinase-HlyAs

Purification step	Total protein (mg)	Total activity (U)	Specific activity (U/mg)	Yield (%)	Purification (fold)
Crude extract	584.9	128497.5	219.7	100	1
Ammonium sulfate fraction	481.7	113334.8	235.3	88.2	1.1
DEAE Sepharose purification	129.9	51369.9	395.5	40.0	1.8
MonoQ purification	68.8	30681.8	446.2	23.9	2.0

### Temperature Optimum and Thermostability of Cutinase-HlyAs

**Figure 3:**
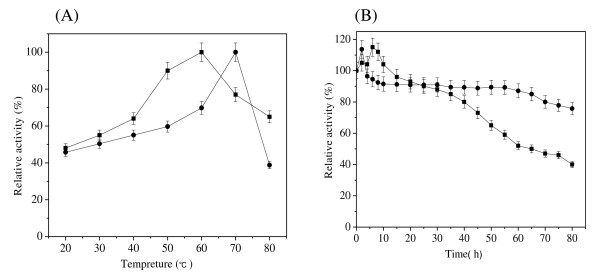
**Effects of temperature on activity and stability of cutinase-HlyAs and cutinase**. (A) Temperature optimum determined in Tris-HCl buffer (pH 8.0); (B) Thermostability of the enzymes in Tris-HCl (pH 8.0) at 50°C. ■, cutinase; ●, cutinase-HlyAs. The error bars show the standard deviations from three measurements.

Recombinant cutinase-HlyAs exhibited optimal activity at 70°C, and the enzyme activity decreased sharply from 70 to 80°C, while cutinase displayed its highest enzyme activity at 60°C (Figure 3a).

The thermostability of cutinase-HlyAs was determined at 50°C. Interestingly, cutinase-HlyAs exhibited greater thermostability than cutinase, with 70% residual activity after incubation for 80 h, while the cutinase showed only 50% residual activity under the same conditions (Figure 3b). The phenomena of this improved thermostability were also found in some other kinds of chimeric proteins [33,34]. Although the mechanism for this improvement is not clear, it may related to protein structure as well as its folding. Further studies are needed to explore this issue.

### pH Optimum and Stability of Cutinase-HlyAs

**Figure 4:**
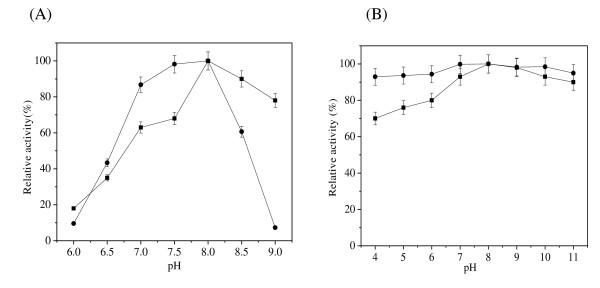
**Effects of pH on activity and stability of cutinase-HlyAs and cutinase**. (A) pH optimum. The samples were determined in the following buffer-potassium phosphate (pH 6.0-7.0) and Tris-HCl (pH 7.0-9.0). (B) pH stability. The samples were assayed after incubation 24 h at 37°C in various buffers-sodium acetate buffer(pH 4.0-6.0), potassium phosphate buffer (pH 6.0-7.0), Tris-HCl buffer (pH 7.0-9.0), and glycine-NaOH buffer (pH 9.0-11.0). ■, cutinase; ●, cutinase-HlyAs. The error bars show the standard deviations from three measurements.

Cutinase-HlyAs exhibited optimal activity at pH 8.0, which is similar to the behavior of cutinase (Figure 4a), although the shapes of their pH-rate curves are somewhat different. Cutinase-HlyAs activity is more sensitive to pH, especially in the alkaline range. A pH-stability investigation was performed from pH 4 to pH 11 (Figure 4b). Cutinase-HlyAs retained more than 90% of its maximal activity after incubation at 37°C for 24 h in the pH range 4-11, which is superior to that of cutinase.

### Cutin Hydrolyzing Activity of Cutinase-HlyAs

In addition to its ability to hydrolyze a short-chain ester (pNPB), cutinase can also catalyze the cleavage of the polyester bonds of cutin, which is composed mainly of C16 and C18 fatty acids. To determine the catalytic efficiency of cutinase-HlyAs toward cutin, both cutinase and cutinase-HlyAs were subject to assay using cutin as substrate. As shown in Table 2 the proportion of C16 and C18 family fatty acid monomers released after enzymatic reaction were 72.13% for cutinase-HlyAs and 71.84% for cutinase. The hydroxy fatty acids, which are the characteristic cutin components, were 1.95% for cutinase-HlyAs and 1.80% for cutinase. These results confirmed that cutinase-HlyAs can hydrolyze cutin as efficiently as cutinase.

**Table 2:** Monomeric products released from cutin hydrolysis by cutinase and cutinase-HlyAs, respectively

Cutin hydrolysis products	Cutinase Hydrolysis Area (%)	Cutinase-HlyAs Hydrolysis Area (%)
Hexadecanoic acid	34.17	31.78
Octadecanoic acid	33.63	35.57
9-Octadecenoic acid	1.16	1.18
9,12-Octadecadienoic acid	1.08	1.65
16-Hydroxy hexadecanoic acid	0.34	0.40
18-Hydroxyoctadeca-9,12-dienoic acid	1.46	1.55

Cutin hydrolysis by cutinase-HlyAs and cutinase were incubated in 25 mM potassium phosphate buffer (pH 8.0) at temperature 50°C for 18 h. The results are the averages of triplicate assays.

### Catalytic Activity of Cutinase-HlyAs toward Cotton fiber/Wettability of Cotton Fabric

Cutinase can improve the wettability of cotton fiber by hydrolyzing the cutin of its waxy cuticle layer, a process referred to as bioscouring. The ability of any particular cutinase preparation to increase wettability is assessed by determining its effect on the wetting time of cotton. To assess the efficacy of cutinase-HlyAs, cotton fiber samples were incubated with either cutinase or cutinase-HlyAs. The wetting times were 15 s for cutinase and 15.8 s for cutinase-HlyAs, while inactivated enzymes showed no effect on the wettability of cotton fabric. HlyAs didn't affect the application of recombinant cutinase in bioscouring.

## Conclusions

In the present study, with concomitant co-expression of HlyB/D, we explored the extracellular expression of *T. fusca *cutinase via α-hemolysin secretion pathway in *E. coli *BL21(DE3). As the expression experiment in the culture media showed, a 334 U/mL of cutinase activity (1.5 mg/mL target proteins) was achieved. In addition, the recombinant protein was purified and the detailed characterization of purified enzyme showed that the recombinant cutinase, even though with c-terminal signal peptide attached, had substrate specificity, pH and temperature profile, as well as application capability in bioscouring similar to that of wild-type cutinase. This is the first report of recombinant *T. fusca *cutinase being efficiently secreted by the α-hemolysin secretion system. Due to the limited cases of successful expression of industrial enzyme by *E. coli *α-hemolysin secretion system, our study further explored the utilization of this pathway in industrial enzymes.

## Methods

### Bacterial Strains, Vectors and Materials

The plasmid cutinase/pET-20b(+), for expressing cutinase through *E. coli *SecB-dependent type II pathway using pelB as signal peptide, was constructed previously [30]. *E. coli *CFT073(ATCC 700928) were purchased from ATCC. *E. coli *JM109 was used as the host for plasmid construction. *E. coli *BL21(DE3) was used as expression host. The *T. fusca *strain was laboratory stock [30]. TOPO TA cloning kit was obtained from Invitrogen. The pMD18-T simple vector and the plasmid pSTV28 were obtained from TakaRa (Dalian, China). The pET-20b(+) vector from Novagen was utilized as expression vector.

### Plasmid Construction

All the primers for gene cloning and plasmid construction are shown in Table 3. The gene encoding cutinase (Tfu_0883, GenBank: YP_288944) lacking its stop codon and N-terminal signal peptide was amplified using plasmid cutinase/pET-20b(+) as template. The gene encoding α-hemolysin specific signal peptide HlyAs (GenBank: NP_755445.1, 965-1024 amino acid residue of HlyA) was amplified using *E. coli *CFT073 genomic DNA as template. Overlapping PCR was utilized to fuse HlyAs to the C-terminal of cutinase using primers cutinase-F, cutinase-HlyAs-F, cutinase-HlyAs-R and HlyAs-R, where underlined bases represented *Nde*I and *Xho*I restriction sites, respectively. The amplification product was gel-purified, ligated into the vector pMD18T-simple to form plasmid cutinase-HlyAs/pMD18-T simple. To eliminate the internal *Nde*I site in HlyAs, site-directed mutagenesis was performed using plasmid cutinase-HlyAs/pMD18-T simple as template, HlyAs-MF and HlyAs-MR as primers, in which the lower-case letters indicate the position of silent mutations. The plasmid with the correctly mutated sequences, which was confirmed by DNA sequencing, was digested with *Nde*I and *Xho*I. The target fragment was then ligated into the similarly restricted expression vector pET-20b(+), resulting in the recombinant plasmid cutinase-HlyAs/pET-20b(+), in which the intrinsic pelB signal peptide in pET-20b(+) vector was eliminated.

**Table 3:** Oligonucleotide primers for the construction process

Name	Primers 5'→3'
cutinase-F	GTAATCCATATGGCCAACCCCTACGAGCGC
cutinase-HlyAs-F	CTCCACCTGCCCGTTCTTAGCCTATGGAAGTC
cutinase-HlyAs-R	GACTTCCATAGGCTAAGAACGGGCAGGTGGAG
HlyAs-R	CCGCTCGAGTTATGCTGATGCTGTCAAAG
HlyAs-MF	GCCAGTGATTTTTCgTATGGACGGAACTC
HlyAs-MR	GAGTTCCGTCGATAcGAAAAATCACTGGC
HlyBD-F	CGGCGAGCTCGGATTCTTGTCATAAAATTG
HlyBD-R	CCACGGATCCTTAACGCTCATGTAAAC

The genes encoding HlyB (GenBank: NP_755448) and HlyD (GenBank: NP_755449) were cloned from *E. coli *CFT073 genomic DNA using primers HlyBD-F and HlyBD-R, where the underlined bases represented *Sac*I and *BamH*I restriction sites. The amplification product was gel-purified, and ligated into PCR^®^2.1-TOPO^® ^vector to form plasmid HlyBD/TOPO. This plasmid was digested with *Sac*I and *BamH*I and ligated into the similarly-restricted expression vector pSTV28, resulting in HlyBD/pSTV28.

The sequences of all of the genes amplified by PCR were confirmed by DNA sequencing.

### Extracellular Expression of *T. fusca *cutinase

*E. coli *BL21(DE3) cells were transformed with cutinase-HlyAs/pET-20b(+) complex with HlyBD/pSTV28. The engineered strains were grown in TB medium, containing 100 μg/ml of ampicillin and 30 μg/ml of chloramphenicol, on a rotary shaker at 37°C until an optical density of 1.5 at 600 nm was reached. Isopropyl-1-thio-β-D-galactopyranoside (IPTG) was added at that point, to a final concentration of 0.4 mM to induce expression. After induction, the culture was grown at 25°C. Cell growth (OD_600_) and enzyme activity were measured at regular intervals.

### Purification of Recombinant Cutinase

The recombinant cutinase-HlyAs was purified as following. The culture medium of the engineering *E. coli *BL21(DE3) was centrifuged at 10,000 × *g *for 20 min at 4°C, and then (NH_4_)_2_SO_4 _was slowly added to the culture supernatant, with stirring, to a final concentration of 70% (w/v). The precipitated protein was collected and dissolved in buffer A (20 mM Tris-HCl, pH 8.0), and then dialyzed against 2 liters of buffer A overnight. The sample was filtered (0.22 μm) and loaded onto a DEAE-Sepharose FF column pre-equilibrated with buffer A. The column was eluted at a flow rate of 1 ml/min with a five-column-volume linear gradient of 0 to 1 M NaCl in buffer A. The fractions containing pNPB hydrolase activity were pooled, and then dialyzed against 1 liter of buffer A at 4°C overnight. The dialyzed sample was subjected to monoQ chromatography using a procedure similar to the one described above. The purified enzyme was concentrated by ultrafiltration (30 kDa cut-off membrane, Amicon) and stored at -20°C.

The recombinant cutinase, which was expressed by SecB-dependent type II pathway in *E. coli *BL21(DE3), was purified according to the previous report [30].

### Enzyme Characterization of Cutinase-HlyAs

Esterase activity, cutinase activity, the optimal temperature/pH, the thermostability and pH stability of cutinase and cutinase-HlyAs were performed as previously described [30,35].

### Catalytic Activity of Cutinase-HlyAs toward Totton fiber/Wettability of Cotton Fabric

For a typical wettability assay, 300 units of enzyme and 0.1 g of cotton fabric were added to 25 mM Tris-HCl, pH 8.0, in a final volume of 40 ml. The mixture was shaken at 200 rpm for 3 h in a water bath pre-equilibrated to the desired temperature. The treated swatches were tested for wettability according to Degani et al [29].

## Competing interests

The authors declare that they have no competing interests.

## Authors' contributions

JW supervised the project, SC designed the study, LQS carried out the experiments and drafted the manuscript, LY and RWW participated in the construction of the plasmids, critically revised and corrected the manuscript, JW and JC provided critical discussion. All authors read and approved the final manuscript.
